# Pioglitazone is effective for multiple phenotyepes of the Zucker fa/fa rat with polycystc ovary morphology and insulin resistance

**DOI:** 10.1186/s13048-018-0395-y

**Published:** 2018-03-27

**Authors:** Miyuki Morishita, Toshiaki Endo, Tsuyoshi Baba, Yoshika Kuno, Keiko Ikeda, Tamotsu Kiya, Hiroyuki Honnma, Tsuyoshi Saito

**Affiliations:** 1 Department of Obstetrics and Gynecology, School of Medicine, Sapporo Medical University, South 1 West 16, Chuo-ku, Sapporo, 060-8543 Japan; 2 Ena Ladies Clinic, South 9-1, Hanakawa, Isikari, 061-3209 Japan; 3 Sapporo ART Clinic, North 7 West 4, Kita-ku, Sapporo, 060-0807 Japan

**Keywords:** PCOS, Polycystic ovary syndrome, PCO, Polycystic ovary, Zucker fa/fa rat, Insulin resistance, Atretic follicle, Pioglitazone, Insulin sensitizer, AMH, Adiponectin

## Abstract

**Background:**

Hyperandrogenism and insulin resistance may be related to the etiology of PCOS. Zucker fa/fa rats with polycystic ovary are obese, have insulin resistance without diabetes mellitus or hyperandrogenism and can be utilized as PCOS model rats without effects of hyperandrogenemia. PCOS patients are reported to have elevated levels of serum anti-Mullerian hormone (AMH), which has an inhibitory action on folliculogenesis, and low levels of serum adiponectin, which blocks apoptosis and induces biological effects in some tissues. Pioglitazone, an insulin sensitizer, is administered to PCOS patients with insulin resistance to induce ovulation but the mechanisms by which this occurs have not been elucidated.

**Methods:**

We purchased 4-week-old female fatty Zucker fa/fa rats as well as lean Zucker +/+ rats for use as control rats with normal insulin sensitivity. The Zucker fa/fa rats were administered pioglitazone (2.5 mg/kg body weight/day) or a vehicle every day for 14 days in separate groups. The Zucker +/+ rats were also administered the vehicle. After 2 weeks of treatment, they were euthanized and we obtained serum samples and both ovaries and determined the body weight, ovarian weight, and serum AMH, adiponectin, testosterone, and androstenedione levels. We also examined ovarian histology to check follicle numbers by using hematoxylin-eosin staining, and the number of atretic follicles using Tdt-mediated dUTP nick end labeling (TUNEL) methods.

**Results:**

The Zucker fa/fa rats used as PCO model rats and Pioglitazone treated PCO model rats were significantly heavier than the Zucker +/+ control rats (*p* < 0.05) at 15 day old. Pioglitazone treatment did not influence body weight or ovarian weight in either group. However, the total number of follicles was significantly larger in the PCO model rats than in the control rats (*P* < 0.05).

Although pioglitazone treatment appeared to decrease the total number of follicles in the PCO model rats, the decrease was not statistically significant. However, pioglitazone treatment significantly decreased the total number of atretic follicles and the rate of atreteic follicles in the PCO model rats (*P* < 0.05). The serum AMH level was significantly higher in the PCO model rats than in the control rats. Pioglitazone treatment significantly decreased the serum AMH level and significantly increased the serum adiponectin level in the PCO model rats (*P* < 0.05). Serum testosterone and androstenedione levels were quite low or undetectable in the 3 groups of rats, and were not influenced by pioglitazone treatment.

**Conclusion:**

In this study, pioglitazone treatment reduced the serum AMH level and increased the serum adiponectin level in PCO model rats. These effects are related to reduction of the total number of atretic follicles and rate of atretic follicles. This proves that pioglitazone treatment improves healthy follicle growth in these PCO model rats with insulin resistance.

## Background

PCOS patients are well known to frequently have insulin resistance. However, it is unknown by what mechanisms insulin resistance induces the pathophysiological condition of PCOS. Thus we need a rat PCOS model with insulin resistance to study their precise relationship. Zucker fatty (fa/fa) rats are a well-understood model of obesity and hyperinsulinemia, which arise when the animals are about 4 weeks of age and gradually worsen thereafter. The cause of the obesity is overeating induced by a loss of leptin signaling associated with a leptin receptor mutation. Recently we proposed these rats as a PCOS model with insulin resistance [1]. These PCO model rats have a higher number follicles than the lean Zucker (+/+) rats used as controls. They also have a higher rate of atretic follicles than the control rats. However, serum androgens are almost undetectable in these rats. Thus, this PCOS model is created by insulin resistance rather than by elevated androgens.

Clinically, insulin sensitizers are often reported to induce ovulation in PCOS patients with anovulation. Pioglitazone is a thiazolidine derivative that is used for the treatment of type 2 diabetes mellitus. It decreases peripheral insulin resistance via the peroxisome proliferator-activated receptor-γ (PPAR-γ) pathway [2]. Several studies have demonstrated that it improves the clinical, hormonal, and metabolic status in patient with PCOS [3–6]. Moreover, this drug directly inhibits estradiol and testosterone production in human ovarian cells in vitro [7]. Pioglitazone counteracts the tumor necrosis factor-α inhibition of FSH-induced follicular development and steroidogenesis in an in vitro mouse preantral follicle culture system [3], and pioglitazone–induced reduction in peripheral insulin resistance and its direct effect on the ovaries might be effective for inducing ovulation in patients with PCOS. A meta-analysis of comparisons of the effects of pioglitazone and metformin in treating patients with PCOS proved that pioglitazone improved the menstrual cycle and ovulation better than metformin [8]. However, the mechanism by which it ameliorates the pathophysiological conditions of PCOS is still unknown.

There are some important serum markers that indicate the status of PCOS such as adiponectin and AMH. Adiponectin is a 30-kDa protein comprised of 247 amino acids in four domains and has been shown to possess insulin-sensitizing [9–11], anti-atherogenic [12, 13] and anti-inflamatory properties [14]. It also reportedly inhibits apoptosis of endothelial cells [15]. Zucker fa/fa rats have a higher serum adiponectin level than lean rats [16]. Adiponectin and its receptors are expressed in the rat testis [17] and ovary [18, 19] and play a significant role in steroidogenesis [20], but the mechanism underlying the changes in ovarian adiponectin expression caused by obesity/insulin resistance remains unknown. Adiponectin has the ability to stimulate insulin sensitivity in many tissues. Although it has multiple biological activities, it was long unclear what effects it had on ovarian function. However, our previous study showed that an abrupt reduction in serum adiponectin appeared to be correlated with acceleration of follicular atresia with aging in mature Zucker fa/fa rats [1].

AMH is also well known to be a Mullerian-inhibiting substance, and it is significantly elevated in the sera of PCOS patients compared to healthy women. AMH has an inhibitory role in the ovary, reducing both primordial follicle initiation and follicle sensitivity to FSH via inhibition of aromatase. Interestingly, Pellatt et al. [21] mentioned in their review article that the reason for the raised AMH level in PCOS might give clues as to the mechanism of anovulation. AMH appears to have a major inhibitory role during folliculogenesis, which may contribute to anovulation in PCOS patients. Therefore we need to clarify the mechanisms by which pioglitazone improves the pathophysiological condition of PCOS using PCO model rats with insulin resistance.

## Methods

### Animal treatments and tissue collection

Animals used in this study were maintained in accordance with the guidelines of the Animal Resources Center of the Sapporo Medical University School of Medicine and the Japan SLC Laboratory Animal Care and Use Committee (D55–0215). Female fatty Zucker fa/fa rats with insulin resistance [22] and lean Zucker +/+ rats with normal insulin sensitivity 4 weeks of age were purchased from the Japan SLC (Shizuoka, Japan).

We administered pioglitazone (2.5 mg/ml in 0.5% methyl cellulose/kg body weight/day) to Zucker fa/fa rats or a vehicle (0.5% methyl cellulose) to Zucker fa/fa rats and Zucker +/+ rats every day for 14 days. After 2 weeks of treatment, the rats were killed and we obtained serum samples and both ovaries. We determined the body weight, ovarian weight, and serum AMH (CLEIA method), adiponectin (latex turbidimetric immunoassay), testosterone (ECLIA method), and androstenedione (RIA precipitation method) levels. We also examined ovarian histology to check follicle numbers by using hematoxylin-eosin staining. Each groups had 5 rats, and totally 15 individual animal were used.

### Tdt-mediated dUTP nick end labeling (TUNEL) methods for atretic follicles

TUNEL was carried out using an ApopTag Plus Peroxidase In Situ Apoptosis Detection Kit (Intergen, NY) as described previously [1]. Tissue samples embedded in paraffin were cut into 5-μm-thick sections, which were then deparaffinized in xylene and rehydrated through a graded ethanol series in distilled water. Each section was first incubated with proteinase K, after which endogenous peroxidase activity was removed by treatment with 0.3% H_2_O_2_ for 20 min at room temperature. After intense washing with distilled water for 15 min, the sections were soaked in TdT buffer for 15 min, and then incubated for 60 min at room temperature in TdT buffer containing biotin. The bionylated dUTP incorporated into the nuclear DNA was reacted with HRP-conjugated streptavidin (1:100 dilution) for 30 min at room temperature, after which the color was developed by immersing the sections for 10 min in 50 mmol/l Tris-HCl buffer (pH 7.6) containing 0.3 mg/ml DAB, 10 mmol imidazole and 0.003% H_2_O_2_. Finally, the sections were counterstained with hematoxylin. Negative controls were reacted in parallel with the omission of the terminal trasferase. For TUNEL assay 2 sections of ovarium were examined from each animals.

### Statistics

Data were analyzed using StatView (Version 4, Abacus Concepts). Quantitative data are presented as mean ± SD. For these experiments, we used two or three groups and made measurements at several different time points. We examined the data of three groups by statistical analysis using Student-Newman-Keuls method or we did a point-by-point comparison between two groups using Mann-Whitney’s test. Values of *P* < 0.05 were considered significant.

## Results

### Body weight and ovarian weight

**Fig. 1:**
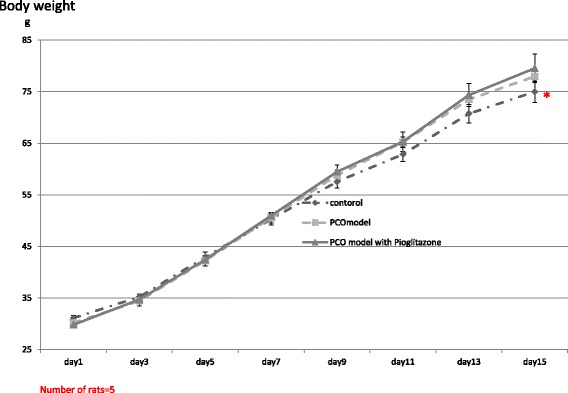
Body weights of control rats (Zucker +/+ rats), PCO model rats (Zucker fa/fa rats) and pitoglitazone-treated PCO model rats, Body weights of these rats were examined every day from day 1(the day pioglitazone injection started) through day 15 (the day the rats were killed). Ovarian weights of these rats were examined on day 15. The weight of PCO model rats and Pioglitazone treated PCO rats model were significantly heavier than that of control rats at day 15. Values are means±SD, *P < 0.05 v.s. PCO model rats, Pioglitazone-treated PCO model rats

**Fig. 2:**
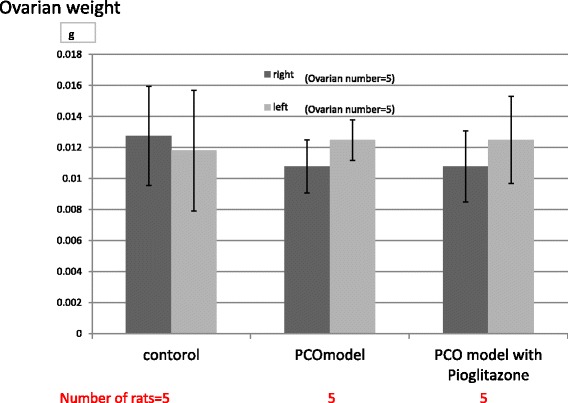
Ovarian weights of control rats (Zucker +/+ rats), PCO model rats (Zucker fa/fa rats) and pitoglitazone-treated PCO model rats. Both ovarian weights of the three groups did not differ. Values are means±SD

The body weights of both PCO model rats and pioglitazone-treated PCO model rats were significantly heavier than for control rats at day 15. There was no significant difference of body weight between the PCO model rats and piogtlitazone-treated PCO model rats (Fig. 1). Ovarian weights of the three groups did not differ (Fig. 2).

### Atretic follicles of ovaries in PCO model rats and pioglitazone-treated rats

**Fig. 3:**
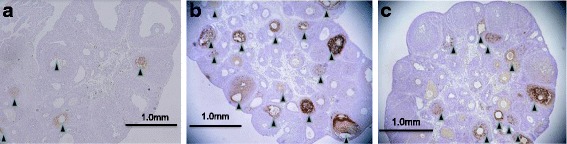
Ovarian histology of atretic follicles detected by the TUNEL method, and total follicle numbers, atretic follicle numbers and rates of atretic follicles of control rats (Zucker +/+ rats), PCO model rats (Zucker fa/fa rats) and pitoglitazone-treated PCO model rats. The TUNEL method and follicle counts of these rats were performed on day 15 in control rats (**a**), PCO model rats (**b**) and Pioglitazone treated PCO model rats (**c**) (the day the rats were killed). Follicles were classified as atretic ▲ if they had five or more apoptic granulosa cells [23]. Values are means±SD, *P < 0.05 v.s. control rats. **P, 0.05 v.s. control rats, Pioglitazone treated PCO model rats

Figure 3a, b and c show representative sections of follicles from the ovaries of rats in the control, PCO model and pioglitazone-treated PCO model rat, respectively. The total number (123.40 ± 11.52) of follicles including preantral follicles and small antral follicles in PCO model rats was significantly larger than in the control rats (90.80 ± 14.50) (*p* < 0.05) as in our previous paper [1]. The total number (100.50 ± 19.95) of follicles of pioglitazone-treated PCO model rats appeared to be lower than in the PCO model rats, but the difference was not significant.

The rates of primodial follicles, primary follicles, and preantral/small antral follicles are generally 15, 20 and 50%, respectively, in all 3 groups. The rate of preovulatory follicles including follicular cysts and corpora lutea was generally less than 15% in all 3 groups. Pioglitazone treatment did not alter the population in any developmental stage of follicles. This prompted us to carry out TUNEL analysis of the follicles and to classify them as atretic if they had five or more apoptic granulosa cells [23]. We found TUNEL-positive cells scattered among the granulosa cells within atretic follicles in both the PCO model rats and control rats; however, the number (65.40 ± 8.65) and rate (52.99 ± 2.85%) of TUNEL-positivity were significantly higher in the small antral/preantral follicles of the PCO model rats than in those (37.25 ± 6.61, 41.19 ± 3.21%) of the control rats (*P* < 0.05). The number and rate of TUNEL-positivity of pioglitazone-treated PCO model rats (44.25 ± 10.28, 43.82 ± 2.78%) were significantly lower than those of the PCO model rats(*P* < 0.05) (Table 1).

**Table 1:** Total follicle number, atretic follicle number and rate of atretic follicles

Number or percent	Control rats	PCO model rats	Pioglitazone treated PCO model rats
Total follicle number	90.80 ± 14.50	123.40 ± 11.52*	100.50 ± 19.95
Atretic follicle number	37.25 ± 6.61	65.40 ± 8.65**	44.25 ± 10.28
Rate of atretic follicle (%)	41.19 ± 3.21%	52.99 ± 2.85%**	43.82 ± 2.78%
Number of rats	5	5	5

Values are mean ± SD

*P < 0.05 v.s. control rats

**P < 0.05 v.s. control rats, Pioglitazone treated PCO model rats

### Hormone profiles of PCO model rats and pioglitazone-treated rats

**Fig. 4:**
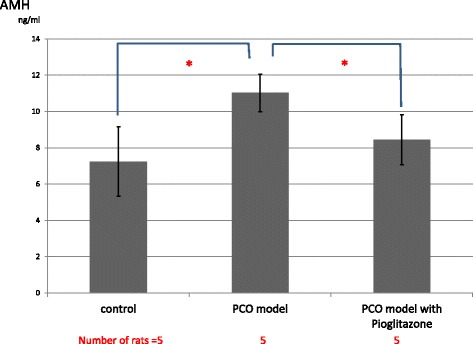
Serum AMH levels of control rats (Zucker +/+ rats), PCO model rats (Zucker fa/fa rats) and pitoglitazone-treated PCO model rats. Serum AMH levels were measured on day 15 (the day the rats were killed). Values are means±SD, *P < 0.05

**Fig. 5:**
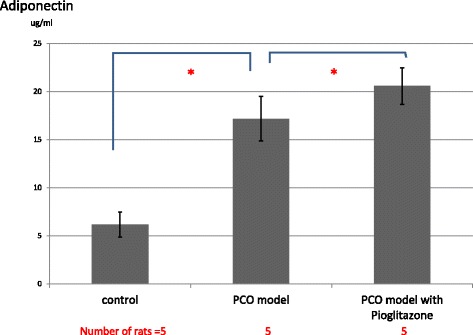
Serum adiponectin levels of control rats (Zucker +/+ rats), PCO model rats (Zucker fa/fa rats) and pitoglitazone-treated PCO model rats. Serum adiponectin levels were measured in these rats on day 15 after fasting the rats for 12 h. Values are means±SD, *P < 0.05

Serum androgens (testosterone and androstenedione) were measured in all 3 groups. However, these androgens were almost under detectable in all 3 groups. The serum AMH levels of PCO model rats were significantly elevated compared to control rats. However, the serum AMH level in pioglitazone-treated PCO model rats was significantly lower than in the vehicle-treated PCO model rats (Fig. 4).The serum adiponectin level in PCO model rats was significantly higher than that in control rats. Moreover, the serum adiponectin level in pioglitazone-treated PCO model rats was significantly higher than in vehicle-treated PCO model rats (Fig. 5).

## Discussion

Our PCO model rats (Zucker fa/fa rats) had a higher number of follicles than the lean Zucker (+/+) rats used as controls, as well as a higher rate of atretic follicles than the control rats. The PCO model rats also had a higher serum AMH level than the control rats as AMH is correlated with the number of follicles. Pioglitazone, which is a selective synthetic agonist of PPAR-gamma, is well known to improve the menstrual cycle and ovulation [8], In this study, pioglitazone treatment reduced the total number and rate of atretic follicles in PCO model rats. Pioglitazone treatment in this rat model also increased the serum adiponectin level. As adiponectin is reported to ameliorate reduced insulin receptor expression and reduce androgen synthesis in ovaries in PCOS [24], the elevated adiponectin level induced by pioglitazone treatment in these model rats possibly ameliorated the pathophysiological status. Pioglitazone treatment also reduced the serum AMH level in the PCO model rats. AMH is speculated to have an inhibitory effect on folliclogenesis in the anovulaton in PCOS. Thus pioglitazone has significant actions on multiple phenotypes of PCO model rats [21]. These results show that pioglitazone might ameliorate the pathophysiological status of PCOS via these multiple actions demonstrated in this study.

PCOS is the commonest endocrine disorder in women, affecting 5–10% of females of reproductive age [25], who account for 30–60% of anovulatory infertility patients. Its basic characteristics are hyperandrogenism, chronic anovulation and polycystic ovaries. Patients very often have insulin resistance, central obesity, impaired glucose tolerance, dyslipidemia, cardiovascular risk and subclinical atherosclerosis [8]. Thiazolines (TZDs) are highly selective synthetic agonists of PPAR-γ used for the treatment of diabetes. The classical insulin sensitizers include troglitazone, rosiglitazone and pioglitazone. The pathogenesis of PCOS may be related to alteration of the PPAR-γ gene, and PPAR-γ seems to play an important role in fertility and metabolism through the effects of its different hypotypes. For example, PPAR-γ can regulate ovarian function [26]. Insulin resistance is an important aspect of PCOS and has been observed not only in obese, but also in lean women with PCOS and seems to be an intrinsic part of the syndrome [27, 28]. PPAR-γ agonists can decrease androgen synthesis in ovaries by ameliorating peripheral insulin resistance indirectly [29]. Thus, there are theoretical and practical bases for the treatment of PCOS with TDZ therapy. The treatment of PCOS with TDZs has been investigated in many animal and clinical studies and trials, and most of the trials showed effective therapeutic results. However, a clear evidence-based explanation of the mechanism by which this occurs is still lacking. In this study, pioglitazone clearly reduced the total number and rate of atretic follicles in PCO model rats, although these effects have not so far been clear in human patients with PCOS. The finding that pioglitazone also reduced serum AMH levels in the PCO rat model supports the above phenomena.

AMH has curious activity in folliculogenesis. Pellatt et al. mentioned in their review article that over the last decade, a new and interesting role has emerged for AMH in the ovary. In human ovaries, AMH is produced by granulosa cells from 36 weeks of gestation until menopause, with the highest expression being in small antral follicles. The AMH production gradually declines as the follicles grow, and once they reach a size at which they are dominant, it has largely disappeared. Its removal from these larger follicles appears to be an important requirement for dominant follicle selection and progression to ovulation as AMH has an inhibitory role in the ovary, reducing both primodial follicle initiation and follicle sensitivity to FSH via inhibition of aromatase [21]. Understanding the reason for the elevated AMH level in PCOS may thus give us an idea of the mechanism of anovulation since AMH appears to have a major inhibitory role during folliculogenesis, which may contribute to anovulation. The effects of pioglitazone treatment in this study provide clues to the mechanisms of pioglitazone treatment for ovulation in the anovulatory status of PCOS via reduction of the high level of serum AMH.

Adiponectin is an adipocytocine that may act in reproduction. Serum adiponectin levels in human PCOS were found to be significantly lower than in controls, suggesting a role for adiponectin in the pathogenesis of the syndrome [30–32]. Adiponectin has several actions in reproduction. It is reported to significantly increase the expression of ovulation-related genes and enhances FSH-mediated induction of *Areg, Has2* and *Ptgs2*, indicating the selective responses of cumulus cells to adiponectin [33]. In cultured theca cells, adiponectin suppresses androstenedione production and gene expression of LH receptors and key enzymes in the androgen synthesis pathway. Moreover, knockdown of genes for AdpoR1 and AdipoR2 is associated with increased androstenedione secretion by bovine theca cells. These results provide evidence for a direct link between fat cell metabolism and ovarian steroidogenesis, suggesting that disruption of adiponectin and/or its receptors plays a key role in the pathogenesis of hyperandrogenenism in PCOS [25]. Recently it was reported that treatment with adiponectin directly ameliorated hyprandrogenemia and increased insulin receptor expression in a PCO-mouse culture experiment [24]. It was also reported that systemic adiponectin treatment increased insulin receptor and restored ovulation [34].

In this study, pioglitazone induced an elevation in serum adiponectin in PCO model rats with insulin resistance, suggesting that it could partially ameliorate the pathological status of PCO via the elevation of adiponectin. The effect of pioglitazone is not due to reduction of fatty tissue, but seems to be a direct action on PPAR-γ because the treatment did not affect the body weight of the rats.

## Conclusions

Pioglitazone treatment is well known to be useful for the anovulation of PCOS patients as noted in a meta-analysis. Unfortunately, the mechanisms for the usefulness of pioglitazone have not hitherto been elucidated. In this study, pioglitazone treatment of PCO model rats reduced the total number and rate of atretic follicles, and also reduced their serum AMH and elevated adiponectin levels. These effects appear to support the usefulness of pioglitazone treatment for PCOS with insulin resistance.

## Abbreviations

AMH: Anti-mullerian hormone
PCO: Polycystic ovary
PCOS: Polycystic ovary syndrome
TNNEL: Tdt-mediated dUTP nick end labeling
